# Effect of Highly Active Antiretroviral Therapy (HAART) and Menopause on Risk of Progression of Cervical Dysplasia in Human Immune-Deficiency Virus- (HIV-) Infected Women

**DOI:** 10.1155/2013/784718

**Published:** 2013-12-18

**Authors:** Suk Chul Kim, Susan Messing, Krupa Shah, Amneris E. Luque

**Affiliations:** ^1^Division of Infectious Diseases, Department of Medicine, University of Rochester Medical Center, P.O. Box 689 601, Elmwood Avenue, Rochester, NY 14642, USA; ^2^Department of Biostatistics and Computational Biology, University of Rochester School of Medicine and Dentistry, Rochester, NY, USA; ^3^Division of Geriatrics and Aging, University of Rochester School of Medicine and Dentistry, Rochester, NY, USA

## Abstract

*Background*. More HIV-infected women are reaching older age and menopause, but there is limited information on cervical squamous intraepithelial lesions (SILs) on these women. *Methods*. To assess the effect of HAART and menopause on SILs in HIV-infected women, we reviewed the results of Papanicolaou (Pap) tests obtained between 1991 and 2011 on 245 women. Progression to SILs was determined by comparing Pap test results. The association of HAART and transition to menopause on SILs was assessed using survival analysis. *Results*. Women receiving HAART had a 52% reduced risk in the progression to SILs compared to women receiving any other antiretroviral regimen or no regimen (CI: 0.33–0.70, *P* = 0.0001). A greater increase of CD4^+^ cell counts was associated with a greater reduction on the risk of progression to SILs. Menopausal women had a 70% higher risk of progression to SILs than premenopausal women (CI: 1.11–2.62, *P* < 0.0001), adjusting for HIV medications, CD4^+^ count, duration of HIV infection, moderation effect of menopause by age, prior IV drug use, and smoking. *Conclusion*. HAART had a positive long-term effect on the progression to SILs. However, being younger and menopausal increases the risk of progression.

## 1. Introduction

HIV infection increases the risk for invasive cervical carcinoma and its precursors, SILs [1–4]. The introduction of HAART has significantly reduced morbidity and mortality in HIV-infected patients. The effect of HAART on SILs, however, has not been entirely clear. Some studies have reported a beneficial effect of HAART with increase in regression [5–8], or decrease in progression [5] of SILs. In contrast, other studies report no difference in regression [9] and progression [9, 10] of SILs, when comparing patients on HAART and those not on HAART. It should be noted that some of these studies had a short-term follow-up and the effect of HAART on SILs may not be obvious because it takes years to develop HPV-related lesions. Two seminal studies done recently showed a definitive beneficial effect of HAART on SIL in HIV-infected women; however, these studies did not assess the effect of menopause [11, 12].

The number of HIV-infected women reaching menopause and older age is expected to increase due to improved survival on HAART. To date, there have only been limited studies which focused on SIL in HIV-infected menopausal women. Therefore, it is not clear whether the general guidelines regarding cervical cancer screening [13, 14] should be applied to HIV-infected menopausal women.

The purpose of this study was to assess the long-term effect of HAART and menopause on SILs in HIV-infected women and to determine the prevalence of SILs in menopausal women with HIV infection.

## 2. Materials and Methods

### 2.1. Study Population

This 20-year retrospective study focused on HIV-infected women who were cared for at Strong Memorial Hospital (SMH) AIDS Center (AC) between January 1991 and December 2011. During this time, SMH AC followed 800–1061 individuals with HIV infection, of which 30% were women. Women were advised to have cervical cytology at baseline and also at 6 months and yearly thereafter if the initial Pap test results were normal. Women with abnormal Pap tests were referred to a gynecology clinic for colposcopy and further management. We had access to the information regarding cytology results and gynecological procedures from both the SMH AC and the gynecology clinic.

In total, 313 female patients were in the SMH AC database and were reviewed. Included in the study were HIV-infected women who were at least 18 years old and had 2 or more cervical Pap tests. This study was approved by the research subjects review board at the University of Rochester.

Women were excluded if they had had a hysterectomy prior to entry into care and/or if they had less than two cervical Pap tests done during the study period. Participants were considered to be postmenopausal as indicated by their clinicians in the medical records. Clinicians at the SMH AIDS Center use the standard definition of 12 months of amenorrhea in the absence of any hormonal therapy [15], and they use levels of follicle-stimulating hormone as supportive criteria as needed [16] to define menopause in HIV-infected women.

Pap test results were reported according to the 1988 and 2001 Bethesda classification [17, 18] as normal, atypical squamous cells of undetermined significance (ASC-US), low grade squamous intraepithelial lesions (LGSIL), high grade squamous intraepithelial lesions (HGSIL), and squamous cell carcinoma. In the 2001 Bethesda system classification [18], atypical squamous cells in which HSILs cannot be excluded (ASC-H) are added in the category of atypical squamous cells. Glandular abnormalities were not included.

For this analysis, we stratified antiretroviral treatments into 4 categories: (1) HAART, (2) “old HAART,” (3) any antiretroviral therapy (“any ART”), and (4) “no ART.” HAART was defined as current standard HAART regimens of two or more nucleoside reverse transcriptase inhibitors (NRTIs) plus at least a boosted protease inhibitor or a nonnucleoside reverse transcriptase inhibitor (NNRTI) or an integrase strand transfer inhibitor (raltegravir) or salvage regimen. “Old HAART” was defined as triple combination antiretroviral regimens containing indinavir, saquinavir, fosamprenavir, nelfinavir, or nevirapine. ART regimens not classified as HAART or “old HAART” were defined as “any ART.” “No ART” indicated that no antiretroviral therapy was used.

Progression to SILs was defined as worsening cervical dysplasia in subsequent Pap test (e.g., normal Pap test changing to ASCUS, LGSIL, HGSIL, or carcinoma in situ or ASCUS changing to LGSIL, HGSIL, or carcinoma in situ). To check on the progression to cervical lesions, two consecutive Pap tests were compared with one another. However, if cervical procedures were done after one Pap test, the Pap test was not compared to the subsequent Pap test. Cervical procedures included punch biopsy, cone biopsy, endocervical curettage, loop electrosurgical excision procedure, or cryotherapy.

Predictors of interest included age, cytology, ethnic group, menopausal status, CD4^+^ cell counts, nadir CD4^+^ cell counts, HIV viral load, HIV medications, risk factors of HIV infection, and history of smoking, and results of Pap tests reported using the 1988 and 2001 Bethesda classification [17, 18]. The number of sexual partners was collected at each visit, but this number may not refer to the number of lifetime sexual partners the patient had.

### 2.2. Statistical Analysis

The Cox proportional hazards model was used to investigate time to progression to SILs allowing for the possibility of a patient having multiple events, and the association of those events with time-dependent covariates (see Table 2), assessed at the visit prior to the event or censoring. To capture potential multiple events, we employed a counting process model which used a partial likelihood determined from the observed survival times of all events, for each subject. The sandwich estimator was used to adjust for the correlation to establish a robust standard error for the parameter estimates.

Following the assessment of the univariate survival models, a backward selection procedure with a *P* > 0.05 was used to determine a parsimonious model from the above-mentioned predictors of interest. All candidate predictors were entered into the model, and at each step, those variables, adjusted for all other variables in the model, that failed to have an associated *P* ≤ 0.05 with the criterion, were removed. The model was evaluated after each variable was removed and the process stopped when all variables remaining in the model had a *P* ≤ 0.05. All analyses used SAS 9.3 software (Copyright © 2011, SAS Institute Inc., Cary, NC, USA) on a Windows 7 platform.

## 3. Results

Among the 313 HIV-infected women in the data base, a total of 68 women were excluded; 38 had a history of hysterectomy, 1 had no available information on antiretroviral therapy, and 29 had fewer than 2 follow-up Pap tests documented during the study period; of these 29 patients, 17 were considered lost to follow-up (less than two cytology results and last visit prior to 2011), and 12 had entered the cohort recently. There was a significant difference in age at entry where those who were lost to follow-up were on average 5 years older than those not lost to follow-up (*P* = 0.0314). While non-Hispanics were more likely than Hispanics and Blacks more likely than Whites to drop out of care (OR = 4.16 (95% CI: 0.54, 32.13) and OR = 1.63 (95% CI: 0.50, 5.33), resp.), the width of the confidence intervals and nonsignificant differences (*P* = 0.1724 and *P* = 0.4182) may reflect the modest numbers that were lost to follow-up.

Two hundred forty five HIV-infected women were eligible for analyses. The number of follow-ups among patients ranged from 2–36 months, and the average number of months between visits over the 245 patients was 11.77 ± 7.10. These women contributed a total of 2,364 cervical tests. The mean number of Pap tests for the cohort was 9.6 with a median of 9 (SD = 6.26), range 2–32, and the prevalence of SIL was 37% at baseline for the entire cohort.  Table 1 shows the baseline characteristics of the cohort.

In the univariate survival analyses (see Table 2), the hazard ratios (HR) revealed that use of HAART, higher CD4^+^ cell counts, menopause, increased duration of HIV infection, and increased age were associated with a decreased hazard of progression to SILs while increased viral load and being a current smoker versus a former smoker were associated with an increased hazard ratio; IV drug use was highly suggestive of a greater hazard of progression. At the visit defined as a progression visit 35% had progressed to ASCUS, 0.37% ASC-H, 57% to LGSIL, 7% to HGSIL, and 0.41% to cancer.

**Figure 1:**
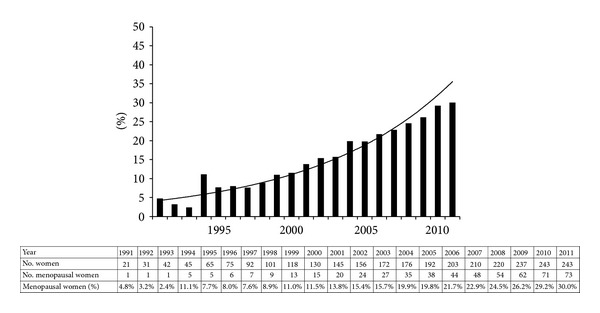
Yearly proportion of menopausal women with HIV infection.

**Figure 2:**
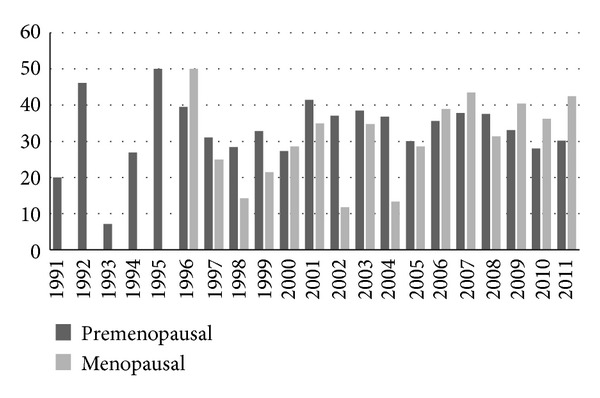
Percentage of SIL by year for premenopausal and menopausal women.

In the multivariate survival analysis (Table 3), antiretroviral therapy, CD4^+^ cell counts, duration of HIV infection, menopause, age, IV drug use, and smoking remained in the model. Use of HAART, higher CD4^+^ cell counts, and increasing age were significantly associated with the lower risk of progression to SILs. Being menopausal was associated with an increased risk of SILs progression contrary to the finding in the univariate analysis. Since being menopausal reduced the hazard of progression in the univariate analysis (Table 2) but increased the hazard in the multivariate analysis we first looked at the variance inflation factor to see whether multicolinearity might have explained this hazard reversal. We did not find evidence of multicolinearity. The other plausible cause for the phenomenon was the potential role of age as a moderator variable, represented by the interaction of age and menopause in the multivariate analysis. In this model, age as a main effect was not a candidate for consideration in the backward elimination procedure since it served as a moderator variable and was highly correlated with menopause [19]. Thus, looking at the contrast of menopause compared to premenopause, the hazard of progression is greater for menopausal women (HR = 1.63, 95% CI = 1.03–2.58, *P* < 0.0001). The interaction term of age by menopause further reveals that HIV-infected women who were menopausal at a younger age evidenced a higher rate of progression to cervical SILs, while the hazard of progression to cervical SILs for older menopausal women was lower (Table 3). No women in our sample were younger than 38 and menopausal. The hazard ratio for the interaction term for those women below 38 and above 55 is essentially an expression of the fitted model. In our study population the average age of menopause was 48.3 ± 4.4. Overall, the proportion of menopausal women has been increasing over time (Figure 1). In 2011, 30% of HIV-infected women were postmenopausal. The prevalence of SILs fluctuated each year and ranged from 8.3% to 50% with a median of 30% and there was no statistically significant difference in the prevalence of SILs in menopausal women compared to nonmenopausal women (Figure 2).

Women on HAART had half the hazard of progression to SILs (HR = 0.47, 95% CI: 0.33–0.68, *P* = 0.0001) when compared to those on any other ART regimen (“old HAART” and “any ART”) and “no ART.” This protection afforded by HAART was maintained when the therapies were looked at separately as well: “old HAART” (HR = 0.46, 95% CI 0.31–0.69, *P* = 0.0002), “any ART” (HR = 0.35, 95% CI 0.19–0.63, *P* = 0.0005), or “no ART” (HR = 0.66, 95% CI 0.47–0.92, *P* = 0.0139). For each 100 cells/*μ*L increase in CD4^+^ cell count, the hazard of progression decreased by 9% (HR = 0.91, 95% CI = 0.86–0.96, *P* = 0.0007). While the *P* value remains the same, the effect of increased CD4^+^ cell counts is more apparent when higher numbers of units of increase in CD4^+^ cell counts are compared. For each year of increase in duration of HIV infection, the hazard of progression decreases by 12% (HR = 0.88, 95% CI, 0.85–0.91, *P* < 0.0001). IV drug use increased the hazard of progression by 94% (HR = 1.94, 95% CI = 1.33–2.84, *P* = 0.0007) as did being a current smoker when compared with those who never smoked (HR = 1.39, 95% CI = 0.99–1.95, *P* = 0.0566) or a former smoker (HR = 2.07, 95% CI = 1.04–4.16, *P* = 0.0397) (Table 3).

## 4. Discussion

The restoration of the immune function mediated by HAART in HIV-infected women may have a positive impact on cervical SILs [20]. Compared to those who are not on HAART, HIV-infected women on antiretroviral therapy tend to have a lower incidence rate of SILs [2] and higher probability of remaining free of SILs [21]. However, the effect of HAART on changes of SILs remains unclear and controversial. In their one-year follow-up study of 2,059 women, Minkoff et al. reported that HIV-infected women who were on HAART had a greater increase in regression and a greater decrease in the progression to SILs compared to women who were not on HAART [5]. It has also been reported that the use of HAART is associated with a higher rate of regression of SILs [6] and CINs [8]. Recently, Adler et al. conducted a large prospective study evaluating the effect of HAART on HPV-related cervical disease; in their study, the use of HAART increased the regression of cervical lesions [7]. The progression to cervical lesions was also reduced by 15% in those on HAART compared to those not on HAART, but the result was not statistically significant [7]. On the other hand, Schuman et al. reported that progression and regression of SILs were not different in HIV-infected women on HAART and not on HAART [9] and Lillo et al. showed no difference in regression of SILs [10]. Firnhaber et al. and Blitz et al. reported in recent studies decreased incidence and progression of SIL in HIV-infected women receiving modern HAART [11, 12].

Our retrospective cohort of prospectively collected information over 20 years aims to bring more clarity to understand the effects of HAART and menopause on the progression to SIL in HIV-infected women. We stratified HAART as two categories: HAART and “old HAART” because ART regimens containing indinavir, saquinavir, fosamprenavir, nelfinavir, or nevirapine are not preferred regimens due to lower potency, side effects, and inconvenient dose schedules [22]. We were able to compare women on HAART to those on “old HAART” because we analyzed up to almost 20 years in cumulative data (median 9.2 (0.4–19.9) years). In our study, we found that HIV-infected women on HAART had a lower risk of progression to SILs. Overall, HAART decreased the progression to cervical SILs by 51% in this stable cohort. Interestingly, we found that “old HAART” did not decrease risk of progression to SILs.

Our findings suggest that the mixed results regarding the effect of HAART on SILs may be due to the lack of stratification by ARV regimen or the use of ARV medications which are considered less potent. In those previous studies [5–10], HAART regimens were probably regimens corresponding to “old HAART” as defined in our study alone or a mixture of “old HAART” and HAART since these studies were conducted between 1993 and 2000 [5, 6, 8–10]. Therefore, it is not surprising that the results of these previous studies were varied. In a recent study by Adler et al. [7], their HAART regimens were not specified and probably included regimens corresponding to HAART as defined in our study.

An increase of CD4^+^ cell counts is a common direct consequence of HAART, and therefore the effect of increased CD4^+^ cell counts on SILs indirectly reflects the effect of HAART. Moore et al. reported that higher CD4^+^ cell counts were associated with higher rates of regression of SILs [23]. Other studies have demonstrated that with lower CD4^+^ cell counts, the prevalence of HPV-related cervical lesions is increased [2]. Our study demonstrates that higher CD4^+^ cell counts are associated with a lower risk of progression to cervical SILs. However, some studies showed a discordant relationship between HAART and CD4^+^ cell count relating with HPV-related cervical lesions. Studies reporting no correlation between HAART and SILs in HIV-infected women demonstrated increased prevalence [10] or risk of progression [9] of SILs with decreased CD4^+^ cell counts. On the other hand, Heard et al. showed positive impact of HAART on cervical SILs but reported that CD4^+^ cell counts were not associated with regression of SILs [8]. These inconsistencies regarding the effect of HAART and CD4^+^ cell counts on SILs on HIV-infected women suggest that additional immunologic factors may play a role in HPV-related cervical disease in suppression HPV [20, 24].

Our study finds that more women reached menopause with substantial improvement of morbidity and mortality in HIV infection after the introduction of HAART. One-third of our study population was menopausal in 2011. To our knowledge, only one study has evaluated prevalence of SILs in menopausal women with HIV infection [25]. Although only 18 patients were included in their study, Ceccaldi et al. also observed a high prevalence of SILs in postmenopausal women with HIV infection (50%) [25]. Our study also finds an increased risk of progression to SILs in menopausal women with HIV infection. Specially, HIV-infected women who are menopausal and younger age are at a much higher risk of progression to SILs than older menopausal women. At 50, the trend is reversed and may reflect reduction of risky behavior or better adjustment to a changing hormonal environment associated with increased age or possibly better adherence to medical advice since they are still part of the cohort.

Our finding of an increased risk of progression to cervical SIL in HIV-infected women who are menopausal at a younger age is important because it has been previously reported that HIV-infected women tend to become menopausal earlier than the general population [26, 27]. In our study, the average age at menopause was lower than that of general population (average age, 48.3 versus 52). In the general population, persistence of HPV infection is higher in women who are 42 or older when compared to younger women [28, 29] and the prevalence of high grade cervical dysplasia and cancer increases with age [29, 30]. Considering that there were only small numbers of high grade SILs in our study, we were not able to assess whether high grade cervical lesions are statistically more prevalent in older HIV-infected women. Although the model suggests that older menopausal women have a reduced hazard of progression, we have not addressed the degree of progression.

There are limitations in our study. Firstly, this is an observational study and as such is limited to the report of the associations that we have found. While additional higher order interactions are of interest, we chose to limit the interactions to the one where the further investigation was necessary (given the change in direction of the hazard ratio in the multivariate analysis when compared to the univariate and our knowledge of the conceptual relationships between these variables), rather than introduce possible spurious associations. Secondly, the analysis in our study was based on events of progression SILs. The second or third progression might have a higher risk of progression compared to the first progression and we did not fit a separate hazard function for each specific event number. However, when we looked at time to first progression, the use of HAART reduced progression to SILs in HIV-infected women (HR, 0.494, 95% CI, 0.334–0.730, *P* = 0.0004). Thirdly, all progressions were treated uniformly in our study. For example, progression from ASCUS to LGSIL or one from HGSIL to cervical cancer is treated as a progression. The beneficial effect of HAART is much less pronounced in advanced SILs either due to failure of restoration of HPV-specific immune response and more genetic changes (e.g., more HPV DNA integration into host DNA) [24]. Notably, there were very few (41 out of 2,364 Pap tests) high grade cervical lesions in our study. Finally, our study did not include information on HPV infection, the main cause of SIL [31]. Although Ahdieh-Grant et al. [6] reported that there was no statistical difference in the status of HPV infection between HIV-infected women with regression of SILs and no regression, Schuman et al. [9] showed that HPV infection is a risk factor related to progression to SILs in HIV-infected women.

In addition, menopausal stage was determined by the patient's clinician and although most clinicians follow standard criteria they may not have applied these criteria uniformly for the entire cohort.

## 5. Conclusion

In conclusion, our study showed that the current standard HAART decreases risk of progression to SILs in HIV-infected women. However, menopausal women with HIV infection have a higher risk of progression to SILs than premenopausal women adjusting for age. Furthermore, HIV-infected women who reach menopause at an early age have the highest risk of progression to cervical SILs. The findings of this study are important because aging with HIV is a critical issue in the developed world and the additive effects of aging, HIV infection, and/or HAART on development or progression to SIL are not well understood. Future prospective studies examining the prevalence, natural course, and characteristics of HPV-related cervical lesions are needed to identify best practice guidelines for treatment and ultimately improve clinical outcomes in this high risk population.

HAART reduces the risk of progression to SILs in HIV-infected women. Menopause is a risk factor to the progression to SILs. HIV-infected women who reach menopause early have higher risk of progression to SILs.

## Figures and Tables

**Table 1:** Baseline characteristics of the cohort.

Baseline characteristics	Total number of women (*n* = 245)
Mean age, years (SD)	36.5 (9.8)
Mean CD4^+^ nadir, cells/mm^3^ (SD)	205.9 (195.3)
Mean baseline HIV RNA, Log_10_ copies/mL (SD)	4.9 log (5.2)
Median number of PAP test (range)	9 (2–32)
Risk factors for HIV infection	
Unprotected heterosexual contact, *n* (%)	205 (83.7%)
IV drug use, *n* (%)	37 (15.1%)
Vertical transmission, *n* (%)	6 (2.4%)
History of smoking	
Current smokers, *n* (%)	131 (53.5%)
Former smokers, *n* (%)	20 (8.2%)
Never smoked, *n* (%)	94 (38.4%)
Race*	
Black, *n* (%)	124 (50.6%)
White, *n* (%)	71 (29.0%)
Other, *n* (%)	50 (20.4%)
Ethnicity	
Hispanic, *n* (%)	48 (19.6%)
Non-Hispanic, *n* (%)	197 (80.4%)

SD: standard deviation.

*Other includes Asians, Native Americans, and Hispanics who declined to identify themselves as black or white.

**Table 2:** Univariate survival analysis of factors associated with progression to cervical SILs.

Variables	Hazard ratio (95% Wald Robust CL)	*P* value
Antiretroviral therapy^§^		
HAART^a^ versus old HAART^b^	0.47 (0.31–0.70)	0.0002
HAART versus any ART^c^	0.33 (0.18–0.61)	0.0004
HAART versus no ART	0.42 (0.30–0.60)	<0.0001
Increased CD4^+^ cell counts (cells/mm^3^)^§∗^		<0.0001
By 100	0.88 (0.82–0.92)	
By 200	0.76 (0.68–0.85)	
By 300	0.66 (0.56–0.78)	
By 500	0.50 (0.36–0.66)	
Increased viral load by 1 log⁡_10_^§^	1.29 (1.21–1.38)	<0.0001
Duration of HIV infection (for each 10 years of increase)^§^	0.30 (0.22–0.41)	<0.0001
Age (for each 10 years of increase)^§^	0.57 (0.49–0.66)	<0.0001
Menopausal versus premenopausal^§^	0.62 (0.41–0.94)	0.0225
Race		0.1891
White versus black	0.78 (0.55–1.10)	0.1580
Hispanic versus non-Hispanic	0.80 (0.54–1.18)	0.2526
Number of sexual partners^§^	1.13 (0.85–1.49)	0.4065
Unprotected heterosexual contact	1.03 (0.62–1.70)	0.9246
HIV infection from IV drug use (yes versus no)	1.46 (1.00–2.13)	0.0516
Smoking status		0.0144
Current smokers versus never smoked	1.30 (0.94–1.77)	0.1208
Current smokers versus former smokers	2.89 (1.32–6.34)	0.0080

HAART: highly active antiretroviral therapy, ART: antiretroviral therapy.

^a^HAART: current standard HAART regimens (two or more nucleoside reverse transcriptase inhibitors (NRTIs) with a protease inhibitor or a nonnucleoside reverse transcriptase inhibitor (NNRTI) or integrase strand transfer inhibitors (raltegravir) or salvage regimen.

^b^Old HAART: triple combination antiretroviral regimens containing indinavir, saquinavir, fosamprenavir, nelfinavir, or nevirapine.

^c^Any ART: not classified as HAART or old HAART.

^§^Time-dependent covariate.

*The different units are presented for the reader's convenience in the incremental units they may prefer.

**Table 3:** Multivariate survival analysis of factors associated with progression to cervical SILs.

Variables	Hazard ratio (95% Wald Robust CL)	*P* value
Antiretroviral therapy		0.0004
HAART^a^ versus old HAART^b^	0.46 (0.31–0.69)	0.0002
HAART versus any ART^c^	0.35 (0.19–0.63)	0.0005
HAART versus no ART	0.66 (0.47–0.918)	0.0139
HAART versus others^d^	0.47 (0.33–0.68)	<0.0001
Increased CD4 counts (cells/mm^3^)		0.0007
By 100	0.91 (0.86–0.96)	
By 200	0.82 (0.74–0.92)	
By 300	0.75 (0.63–0.89)	
By 500	0.62 (0.47–0.82)	
Duration of HIV infection (by 1 year)	0.88 (0.85–0.91)	<0.0001
Menopausal versus premenopausal (at mean age = 40.87)	1.63 (1.03–2.58)	<0.0001
Age and menopause interaction*		<0.0001
Meno yes versus no at age = 30	3.62 (1.83–7.17)	<0.0005
Meno yes versus no at age = 40	1.74 (1.08–2.79)	<0.0050
Meno yes versus no at age = 50	0.83 (0.57–1.22)	>0.5000
Meno yes versus no at age = 60	0.40 (0.25–0.64)	<0.0005
HIV infection from IV drug use (yes versus no)	1.94 (1.33–2.84)	0.0007
Smoking status		0.0306
Current smokers versus never smoked	1.39 (0.99–1.95)	0.0566
Current smokers versus former smokers	2.07 (1.04–4.16)	0.0397

HAART: highly active antiretroviral therapy, ART: antiretroviral therapy.

^a^HAART: current standard HAART regimens (two or more nucleoside reverse transcriptase inhibitors (NRTIs) with a protease inhibitor or a nonnucleoside reverse transcriptase inhibitor (NNRTI) or integrase strand transfer inhibitors (raltegravir) or salvage regimen.

^b^Old HAART: triple combination antiretroviral regimens containing indinavir, saquinavir, fosamprenavir, nelfinavir, or nevirapine.

^c^Any ART: not classified as HAART or old HAART.

^d^Others: ART regimen except HAART (“old HAART” and “any ART”) and “no ART”.

*P values are approximations and ages are extrapolations from the regression equation.

## References

[1] Engels EA, Biggar RJ, Hall HI (2008). Cancer risk in people infected with human immunodeficiency virus in the United States. *International Journal of Cancer*.

[2] Delmas MC, Larsen C, van Benthem B (2000). Cervical squamous intraepithelial lesions in HIV-infected women: prevalence, incidence and regression. *AIDS*.

[3] Kadhel P, Multigner L, Bardinet F, Goerger-Sow MT, Janky E (2012). Cervical intraepithelial neoplasia and invasive cancer risks in women infected with HIV in the French West Indies. *HIV Medicine*.

[4] Ellerbrock TV, Chiasson MA, Bush TJ (2000). Incidence of cervical squamous intraepithelial lesions in HIV-infected women. *Journal of the American Medical Association*.

[5] Minkoff H, Ahdieh L, Massad LS (2001). The effect of highly active antiretroviral therapy on cervical cytologic changes associated with oncogenic HPV among HIV-infected women. *AIDS*.

[6] Ahdieh-Grant L, Li R, Levine AM (2004). Highly active antiretroviral therapy and cervical squamous intraepithelial lesions in human immunodeficiency virus-positive women. *Journal of the National Cancer Institute*.

[7] Adler DH, Kakinami L, Modisenyane T (2012). Increased regression and decreased incidence of HPV-related cervical lesions among HIV-infected women on HAART. *AIDS*.

[8] Heard I, Tassie J-M, Kazatchkine MD, Orth G (2002). Highly active antiretroviral therapy enhances regression of cervical intraepithelial neoplasia in HIV-seropositive women. *AIDS*.

[9] Schuman P, Ohmit SE, Klein RS (2003). Longitudinal study of cervical squamous intraepithelial lesions in Human Immunodefidency Virus (HIV)-seropositive and at-risk HIV-seronegative women. *The Journal of Infectious Diseases*.

[10] Lillo FB, Ferrari D, Veglia F (2001). Human papillomavirus infection and associated cervical disease in human immunodeficiency virus-infected women: effect of highly active antiretroviral therapy. *The Journal of Infectious Diseases*.

[11] Blitz S, Baxter J, Raboud J (2013). Evaluation of HIV and highly active antiretroviral therapy on the natural history of human papillomavirus infection and cervical cytopathologic findings in HIV-positive and high-risk HIV-negative women. *The Journal of Infectious Diseases*.

[12] Firnhaber C, Westreich D, Schulze D (2012). Highly active antiretroviral therapy and cervical dysplasia in HIV-positive women in South Africa. *Journal of the International AIDS Society*.

[13] ACOG Committee on Practice Bulletins—Gynecology (2009). ACOG practice bulletin no. 109: cervical cytology screening. *Obstetrics and Gynecology*.

[14] Bernstein R, Dejoseph D, Buchanan EM (2010). When to stop screening: a review of breast, gynecologic, and colorectal cancer screening in women over age 65. *Care Management Journals*.

[15] Kanapathipillai R, Hickey M, Giles M (2013). Human immunodeficiency virus and menopause. *Menopause*.

[16] Harlow SD, Gass M, Hall JE (2012). Executive summary of the stages of reproductive aging workshop + 10: addressing the unfinished agenda of staging reproductive aging. *Journal of Clinical Endocrinology and Metabolism*.

[17] (1989). The 1988 Bethesda system for reporting cervical/vaginal cytological diagnoses. National Cancer Institute Workshop. *The Journal of the American Medical Association*.

[18] Solomon D, Davey D, Kurman R (2002). The 2001 bethesda system: terminology for reporting results of cervical cytology. *The Journal of the American Medical Association*.

[19] Baron RM, Kenny DA (1986). The moderator-mediator variable distinction in social psychological research. Conceptual, strategic, and statistical considerations. *Journal of Personality and Social Psychology*.

[20] Adler DH (2010). The impact of HAART on HPV-related cervical disease. *Current HIV research*.

[21] Sirera G, Videla S, López-Blázquez R (2008). Highly active antiretroviral therapy and incidence of cervical squamous intraepithelial lesions among HIV-infected women with normal cytology and CD4 counts above 350 cells/mm^3^. *Journal of Antimicrobial Chemotherapy*.

[22] Panel on antiretroviral guidelines for adults and adolescents. Guidelines for the use of antiretroviral agents in HIV-1-infected adults and adolescents. http://www.aidsinfo.nih.gov/ContentFiles/AdultandAdolescentGL.pdf.

[23] Moore AL, Sabin CA, Madge S, Mocroft A, Reid W, Johnson MA (2002). Highly active antiretroviral therapy and cervical intraepithelial neoplasia. *AIDS*.

[24] Palefsky J (2006). Biology of HPV in HIV infection. *Advances in Dental Research*.

[25] Ceccaldi PF, Ferreira C, Coussy F (2010). Cervical disease in postmenopausal HIV-1-infected women. *Journal de Gynécologie, Obstétrique et Biologie de la Reproduction*.

[26] Massad LS, Evans CT, Minkoff H (2006). Effects of HIV infection and its treatment on self-reported menstrual abnormalities in women. *Journal of Women’s Health*.

[27] Schoenbaum EE, Hartel D, Lo Y (2005). HIV infection, drug use, and onset of natural menopause. *Clinical Infectious Diseases*.

[28] Rodriguez AC, Schiffman M, Herrero R (2010). Longitudinal study of human papillomavirus persistence and cervical intraepithelial neoplasia grade 2/3: critical role of duration of infection. *Journal of the National Cancer Institute*.

[29] Ansari M, Mehdi G, Arif SH, Ansari H, Khan T (2012). Smear patterns and spectrum of premalignant and malignant cervical epithelial lesions in postmenopausal Indian women: a hospital-based study. *Diagnostic Cytopathology*.

[30] Moore KN, Bannon RJ, Lanneau GS, Zuna RE, Walker JL, Gold MA (2008). Cervical dysplasia among women over 35 years of age. *American Journal of Obstetrics and Gynecology*.

[31] Centers for Disease Control and Prevention Cervical cancer. http://www.cdc.gov/Cancer/Cervical.

